# Dynamic, multi‐scale analyses indicate site‐ and landscape‐level forest cover drive Yellow‐billed and Black‐billed Cuckoo interannual turnover

**DOI:** 10.1002/ece3.10938

**Published:** 2024-02-07

**Authors:** Claire A. Johnson, Thomas J. Benson

**Affiliations:** ^1^ Illinois Natural History Survey, Prairie Research Institute University of Illinois, Urbana‐Champaign Champaign Illinois USA; ^2^ Department of Natural Resources and Environmental Sciences University of Illinois, Urbana‐Champaign Urbana Illinois USA

**Keywords:** Black‐billed Cuckoo, habitat use, multi‐scale, occupancy dynamics, turnover, Yellow‐billed Cuckoo

## Abstract

Studies of habitat use in breeding birds often assume species have relatively stable breeding distributions. Some species, however, display considerable year‐to‐year variability, complicating efforts to determine suitable or preferred habitats. After returning to their breeding range, Black‐billed Cuckoos (*Coccyzus erythropthalmus*) and Yellow‐billed Cuckoos (*C. americanus*) are thought to range widely before nesting, resulting in high rates of interannual breeding‐site turnover, potentially contributing to conflicting habitat associations found in past studies. However, difficulty detecting these rare and declining species could lead to overinflated estimates of interannual turnover. Using broadcast surveys to increase detection probability, we collected detection/non‐detection data in 2019 and 2020 at 41 publicly owned sites in Illinois and performed a dynamic, multi‐scale occupancy analysis for each species to separate detection probability from potential interannual turnover and determine landscape and small‐scale variables driving habitat use and occupancy dynamics. We found strong support for interannual turnover for both species based on poor performance of non‐dynamic models and variation in estimated annual occupancy (20% and 21% increase between years for Black‐billed and Yellow‐billed Cuckoos, respectively). Black‐billed Cuckoos persisted at sites with less forest in the surrounding landscape and used areas with denser understory vegetation. Yellow‐billed Cuckoos colonized sites with greater canopy cover, avoided developed landscapes, and used areas with a shorter subcanopy layer. The dynamic nature of habitat use in these two cuckoo species suggests the importance of coordinating management and conservation across a broader spatial scale. Managing for larger patches of dense shrubs in less forested landscapes would benefit Black‐billed Cuckoos while Yellow‐billed cuckoos would benefit from management creating forested areas with open understories in less‐developed landscapes.

## Graphical abstract

Both Black‐billed and Yellow‐billed Cuckoos showed significant site‐level turnover between years. Black‐billed Cuckoos were associated with greater vertical vegetation cover within sites and persisted at sites with less forest cover in the surrounding landscape, while Yellow‐billed Cuckoos used areas with shorter or absent subcanopy layers within less‐developed landscapes. Sites with greater canopy cover were more likely to be colonized by Yellow‐billed Cuckoos.

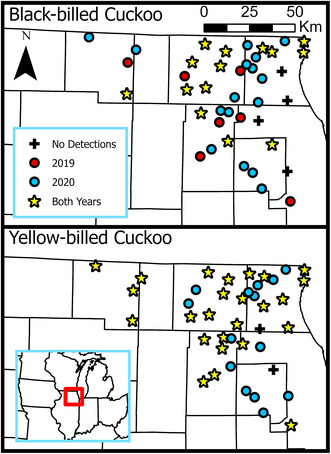

## 1 INTRODUCTION

Determining habitat requirements and appropriate conservation action is difficult for hard‐to‐detect and rare species (Beissinger et al., 2000), more so if they are nomadic or prone to between‐year breeding‐season dispersal (Cottee‐Jones et al., 2016; Greenwood & Harvey, 1982; Teitelbaum & Mueller, 2019). For these species, annual distributions and local populations can be highly variable (Green et al., 2018; Schlossberg, 2009), with some species occupying vastly different areas from year to year (Webb et al., 2017). Generally, intermittent use of a location is assumed to indicate habitat is of low quality (Battin, 2004). However, for species with variable distributions, this assumption could result in erroneous conservation and management decisions (Green et al., 2018). Furthermore, longer‐distance movements also have implications for population dynamics, range expansion potential, and extinction risk (Ponchon et al., 2015). As such, identifying and accounting for movement strategies is necessary for understanding both distributional changes and intensity of habitat selection (MacKenzie et al., 2003; Schlossberg & King, 2007).

Importantly, occupancy dynamics may be influenced by habitat variables at multiple spatial scales (Wiens, 1989). For example, responses to specific variables may depend on the scale at which the variable is measured (Hagen et al., 2016), and in some cases, these responses may be in opposite directions (Chiavacci et al., 2018; Sherry & Holmes, 1988). In addition to providing clarity about species' ecology, scale‐specific information may help inform more effective conservation and management decisions. Accounting for landscape context can help prioritize protecting areas that are most likely to benefit a species of interest, while local habitat relationships can guide management strategies within a protected area (Duren et al., 2011; Green et al., 2018). Failing to consider multiple scales could sacrifice these important insights, especially for dispersal prone or nomadic species, as they may be influenced by spatial extent in different ways than more sedentary species (Wiens, 1989).

Black‐billed and Yellow‐billed Cuckoos (*Coccyzus erythropthalmus*, *C. americanus*) are secretive and hard to detect (Hughes, 2020a, 2020b), and broadscale surveys suggest the populations of both species have declined over the past several decades (Sauer et al., 2020). Our understanding of cuckoo habitat requirements is limited, with studies sometimes indicating conflicting habitat relationships for both species. For example, Black billed‐Cuckoos are associated with larger tracts of deciduous forest and wooded wetlands in some parts of their range (Thogmartin & Knutson, 2007) but are strongly linked to shrublands and thickets in other areas (Robinson et al., 1999). Yellow‐billed Cuckoos are associated with larger forested areas with high tree density (Reiley & Benson, 2019), floodplain forests (McClure & Hill, 2012), and can also be found in brushy fields with patches of small trees (Nolan, 1963). As outlined above, conflicting habitat relationships may be the result of Black‐billed and Yellow‐billed Cuckoos having variable annual distributions. There is evidence that these two species, and others in the genus *Coccyzus*, are highly mobile, even nomadic, and are prone to dispersing both within and between breeding seasons (Barber, 2008; Gale et al., 2001; Halterman, 2009; Lloyd, 2017; Sechrist et al., 2012). This could result in locations changing from occupied to unoccupied (extinction), or unoccupied to occupied (colonization) over a given timeframe (e.g., turnover). For rare and secretive species like cuckoos, a dynamic occupancy modeling framework could provide insight into movement ecology and habitat use. Dynamic models estimate the probability of a state change (e.g., unoccupied to occupied) in addition to quantifying occupancy probability and accounting for imperfect detection (MacKenzie et al., 2003). Accounting for detection is especially important in this context so that false negative detections (an individual is available but not detected) are not mistaken for turnover (an individual is not available for detection; Stanislav et al., 2010). Extending this model to examine multiple spatial scales could provide even more information about cuckoo habitat requirements and the importance of landscape context.

In an effort to both expand our knowledge and provide insights into the conservation and management of Black‐billed and Yellow‐billed Cuckoos, we examined habitat requirements and interannual dynamics of these two species. Given evidence from prior research, specifically in our study region (e.g., Robinson et al., 1999; Wilson, 1999), we expected that Black‐billed Cuckoos would be associated with shrubland habitat within study sites, and open habitat in the landscape and that Yellow‐billed Cuckoos would be associated with forest habitat at both small and large scales. We also expected that both species would demonstrate turnover between years (Gale et al., 2001). We conducted surveys at 41 sites in northeastern Illinois across 2 breeding seasons to examine occupancy and turnover, using call broadcasts to overcome issues of low detection (MacKenzie et al., 2002; Thogmartin et al., 2006). Using dynamic, multi‐scale occupancy models, we explored how landscape context and local habitat relationships affect occupancy and habitat use, and tested for the presence of interannual turnover and habitat variables that might drive state changes.

## 2 METHODS

### 2.1 Study area

**FIGURE 1:**
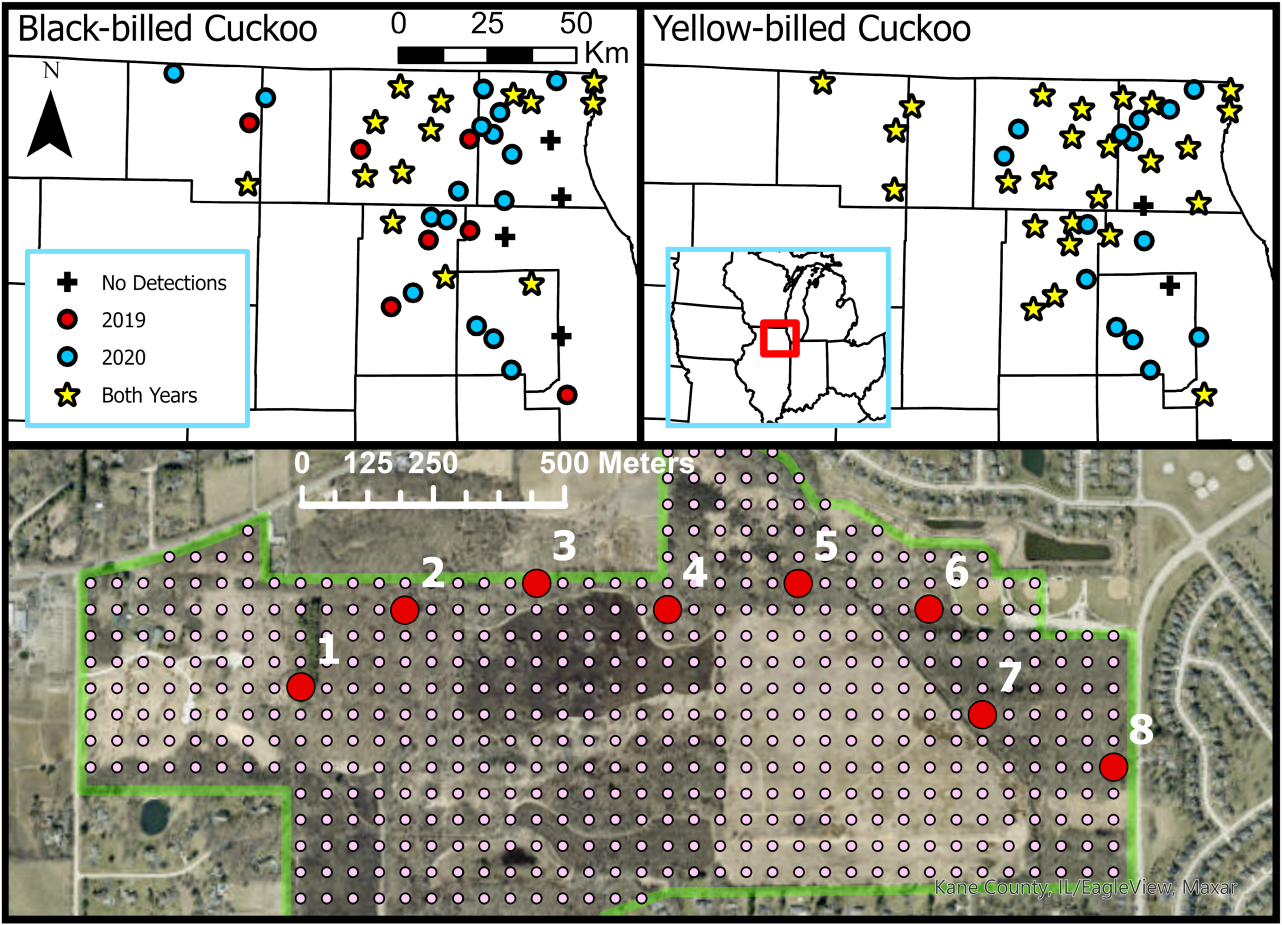
We surveyed 41 sites on public lands in northeastern Illinois and recorded Black‐billed and Yellow‐billed Cuckoo detections in 2019 and 2020. Illinois counties are outlined in black. Within sites (boundary shown in green), survey points (red) were selected from a 50‐m grid (pink) and progressed through the longest axis of potential habitat.

We conducted surveys for Black‐billed and Yellow‐billed Cuckoos throughout northeastern Illinois, USA. Potential study sites were selected from the pool of lands owned by the Illinois Department of Natural Resources or county conservation districts that were open to the public in Boone, Cook, DuPage, Kane, Lake, McHenry, and Winnebago counties (Figure 1). From these, we selected only properties that contained a high proportion of shrubland, edge, and forest habitat, habitat types thought to be used by both cuckoo species in our study region (Graber & Graber, 1963; Robinson et al., 1999; Wilson, 1999). We further reduced the number of potential sites by considering driving time from a central field location, setting a minimum distance between properties (4 km, median 8.4 km), and including a proportion of sites that had a previous record of Black‐billed Cuckoo presence. While both cuckoos breed in this region, Black‐billed Cuckoos are uncommon in Illinois (Kleen et al., 2004). Therefore, we selected two‐thirds of sites to have had at least one Black‐billed Cuckoo observation within the last 10 years (eBird, 2021) to increase the chances of sampling potentially suitable habitat. We looked at occurrences over the previous decade because Black‐billed Cuckoos were only sporadically documented at most locations. The final 41 sites we selected ranged from 68 to 1985 ha but were often near or contiguous to other potential habitat, and the landscape surrounding sites was comprised of a diversity of landcover types.

We used Geographic Information Systems (GISs) to overlay each of our sites with a 50‐m grid from which we selected a transect of points. The transect progressed through the longest axis of potential habitat at the site, including forest, shrubland, and edge, with the starting point located within potential habitat and close to a safe vehicle pull off (Figure 1). Transect length depended on site size, and points were spaced 250 m apart, the upper limit at which a Yellow‐billed Cuckoo could be detected (Halterman, 2009). We selected 345 total points across the study region, and individual transects contained between 4 and 11 points, with an average of 8.4 points (±0.2 SE).

### 2.2 Point count and broadcast surveys

We conducted surveys between 15 min prior to and 4 h after local sunrise. Before starting surveys at each point, we recorded wind speed (Beaufort scale), cloud cover (0%–25%, 26%–50%, 51%–75%, 76%–100%), temperature, and noise level (proportion of survey time during which hearing was significantly impaired; 0%, 1%–25%, 26%–50%, 51%–75%, 76%–100%), treating these as continuous, ordinal variables. We did not survey in steady rain or high winds (>5 on the Beaufort scale) and did not initiate surveys during temporarily noisy conditions (e.g., planes overhead).

At odd‐numbered transect points, we performed broadcast surveys for Black‐billed and Yellow‐billed Cuckoos. At even‐numbered points, we conducted a 5‐min, unlimited radius, passive point count, followed by a broadcast survey. Broadcast surveys for each species were split into three, 1‐min intervals, during which we played roughly 30 s of species‐specific vocalizations (Driver, 2005; Lane, 2005; Nelson 2004a, 2004b, 2004c) followed by 30 s of listening. Species broadcast order was randomly selected at each point and included 1 min of silence between species broadcasts to limit effects due to heterospecific interactions. We did not record detections during the silent period and stopped broadcasting once the focal species was detected to help prevent cuckoos from following surveyors to subsequent points. We broadcast recordings using FOXPRO Firestorm game callers (FOXPRO, Lewiston, PA, USA) at a standardized volume to primarily elicit responses from nearby individuals (<100 m). We surveyed all sites two to three times between May 15, 2019 and August 15, 2020 with survey rounds ≥3 weeks apart.

### 2.3 Vegetation sampling

We sampled vegetation using a modified BBIRD protocol (Martin et al., 1997). Prior research suggests that Yellow‐billed Cuckoos use younger, denser forest plots, forage in taller vegetation (>10 m), and nest in areas with greater canopy cover (Laymon & Halterman, 1985; Reiley & Benson, 2019; Wilson, 1999), while Black‐billed Cuckoos nest in clumps of shrubs or young trees (Robinson et al., 1999). Thus, we were primarily interested in the characteristics of woody vegetation cover and composition. Given environmental conditions were similar in 2019 and 2020, we considered woody cover to be relatively stable following tree leaf out (around June 1), so we conducted sampling once over the course of the study between June 1 and July 30, 2019, only resampling in 2020 if habitat management resulted in significant changes in woody cover (e.g., brush removal or prescribed burning).

We measured the following within an 11.3‐m plot centered at the survey point unless otherwise noted: (1) canopy cover, measured using a densiometer and averaged across readings taken in the four cardinal directions; (2) tree canopy height (average maximum height of woody vegetation >6 m); (3) subcanopy height (average maximum height of woody vegetation ≤6 m); (4) a number of small trees (8–23 cm diameter at breast height [DBH]); (5) a number of medium trees (23–38 cm DBH); (6) shrub cover (proportion of the 5‐m plot covered by living woody vegetation including brambles and saplings, ≤50 cm); (7) brush cover (proportion of the 5‐m plot covered by dead woody vegetation, standing or fallen over, ≤50 cm); and (8) vertical vegetation cover. We visually estimated vertical vegetation cover between 0 and 2 m using a 2‐m tall, 30.5‐cm wide cover board (Nudds, 1977), recording the proportion of the board covered by vegetation when standing 11.3 m away. We took a measurement in the 4 cardinal directions and averaged these readings into a single value.

### 2.4 Spatial analyses

We explored how landscape context affects initial site‐level occupancy and annual dynamics using GIS. We used the 2016 National Landcover Database (NLCD) from the Multi‐Resolution Land Characteristics Consortium (Jin et al., 2019) to determine the proportion of landcover classes within a 700‐m buffer of each transect, an area much larger than the average Yellow‐billed Cuckoo home range (Halterman, 2009). We were especially interested in shrubland availability, as this is an important nesting habitat for both species (Hughes, 2020a, 2020b). However, due to frequent misclassification (Wickham et al., 2017), we combined the shrub/scrub class with herbaceous and hay/pasture into an “open habitat” class as a proxy for early successional, edgy habitat. We also considered the amount of developed area (developed open space and low, medium, and high development), cultivated crops, and forest (deciduous, evergreen, mixed forest, and woody wetlands).

### 2.5 Statistical analyses

Dynamic, multi‐scale occupancy models are an extension of multi‐scale models, which can easily accommodate different combinations of spatial and temporal replication (e.g., Nichols et al., 2008; Pavlacky et al., 2012). Our dynamic, multi‐scale setup was similar to that used by Green et al. (2018) and Tingley et al. (2018) for other bird species. In our analysis, the multi‐scale aspect allowed for the estimation of initial large‐scale occupancy (occupancy of a transect, henceforth *site level*):

$$\psi_{1}=\mathrm{Pr}\;\left(\text{site occupied in year}\;1\right)$$

and small‐scale occupancy (occupancy of a point), conditional on the larger unit being occupied:

$$\theta_{t}=\mathrm{Pr}\;\left(\text{species is present}\;\mathrm{at}\;a\;\text{point in year}\;t\;|\;\text{site is occupied in year}\;t\right)$$

similar to Nichols et al. (2008). Given that cuckoos could become temporarily unavailable for detection at a given survey point during the season, we refer to the small‐scale parameter as point‐level use to relax the assumption of within‐season closure (Kéry & Royle, 2021). This spatially nested aspect also accounts for potential non‐independence among transect points (Kéry & Royle, 2016; Nichols et al., 2008). The dynamic portion of the model allowed for the estimation of colonization (*γ*; probability that a site changes from unoccupied to occupied between years) and extinction (*ε*; probability that a site changes from occupied to unoccupied between years) of large sample units between primary sample periods (MacKenzie et al., 2003). Extinction and colonization can be used to estimate site‐level occupancy probability in subsequent years:

$$\psi_{t}=\psi_{\left(t-1\right)}\times \left(1-\varepsilon_{\left(t-1\right)}\right)+\left(1-\psi_{\left(t-1\right)}\right)\times \gamma_{\left(t-1\right)}$$

Broadcast surveys greatly increase detection compared to passive surveys for both cuckoo species (Johnson & Benson, 2022). Thus, we reduced our encounter history to whether a species was detected at a point during *either* the passive or broadcast survey during a given survey round, and used replicate surveys at a point to estimate detection probability:

$$\begin{array}{c} p_{\mathrm{kt}}=\mathrm{Pr}\;(\text{species detected}\;\mathrm{at}\;a\;\text{point}\;\mathrm{on}\;\text{survey round}\;k\;\text{in year}\;t\;|\\ \;\text{species is present}\;\mathrm{at}\;\text{the point in year}\;t\;\text{and the site is occupied in year}\;t) \end{array}$$

As an example of our data structure, for a site with two survey points, an encounter history of 100 001 000 000 would indicate an individual was detected at point 1 during the first survey round of year 1, at point 2 during the third survey round of year 1, and was never detected at either point in year 2. We performed similar analyses for both Black‐billed and Yellow‐billed Cuckoos.

We limited the number of models under consideration using a sequential‐by‐sub‐model process (Morin et al., 2020). Models were constructed in Program MARK 9.0 using the “Robust Design Multi‐scale occupancy estimation” data type (White & Burnham, 1999) and ranked using Akaike's Information Criterion with a correction for small sample sizes (AIC_c_; Burnham & Anderson, 2002). We first found the best covariate structure for detection probability, with no covariates included in the structures for the other four parameters. We then carried the best detection structure forward to subsequent models. We modeled initial site‐level occupancy next, followed by point‐level use, given it is dependent on the occupancy of the larger sample unit. To model colonization and extinction, we created a separate sub‐model set for each parameter, as there was no definitive reason to model one before the other. We identified well‐supported structures from these two sub‐model sets (ΔAIC_c_ ≤ 2) to use in the final model set, creating models with every combination of the supported colonization and extinction structures. In each sub‐model set, we included a model with the parameter of interest held constant to assess whether adding covariates explained more variation in the data than the constant model. In the final model set, we also included a model with the best structure for detection, initial site‐level occupancy, and point‐level use, but constrained colonization and emigration to zero. By including this non‐dynamic model, we were able to test for the presence of interannual turnover (Betts et al., 2008; MacKenzie et al., 2003). We interpreted coefficients with 85% confidence intervals that did not overlap zero as being strongly supported to stay consistent with the significance threshold used for AIC model selection (Arnold, 2010). We also considered model rank and weight (*w*_*i*_) to determine the importance of individual habitat covariates.

To model detection probability, we first considered the importance of within‐season temporal variation (survey round; Wilson & Bart, 1985) and survey year. We considered the individual effects of both, carrying the top model forward to examine the additive effects of wind, cloud cover, temperature, or noise. For initial site‐level occupancy, we considered single‐variable models of the 8 vegetation variables, averaged by site, and the 4 landscape variables. For point‐level use, we first assessed the importance of year effects, and if the year was well supported, included it in additive models with each of the 8 vegetation variables. We considered site‐averaged vegetation and landscape composition variables in the colonization and extinction sub‐model sets. All covariates were scaled prior to analysis, but we present intercept and coefficient (*β*) values on the original logit scale of each covariate to help with interpreting their biological effect. Covariates are summarized in Table S1.

Colonization and extinction parameters were estimated near the boundaries of 1 or 0 with especially large or small standard errors in a few cases, indicating potential estimation problems using the default optimization method. In these cases, we followed the default approach by using simulated annealing (Goffe et al., 1994) to re‐estimate all models in the problematic sub‐model set prior to selecting a model to carry forward, then used annealing for the rest of the model selection process, taking care to ensure that coefficient values were within a range suitable for this approach. If needed, we used a Markov Chain Monte Carlo (MCMC) approach available in Program MARK to further improve the precision of parameter estimates and the ability to make inferences from the top model. When using MCMC, we estimated model parameters using normally distributed, uninformative priors on the logit scale (Mean = 0, *σ* = 1.75) with initial parameter estimates from the top model generated using simulated annealing. We ran 3 chains with 10,000 tuning, 10,000 burn‐in, and 50,000 stored samples and no thinning. The Gelman‐Rubin statistic indicated convergence for all parameters (R‐hat ≤1; Gelman et al., 2003).

## 3 RESULTS

We surveyed all 41 sites 3 times in both years, except in 2019 when four sites were only surveyed twice. All surveys were included in our analyses. We completed 993 surveys in 2019 and 1029 in 2020. Black‐billed Cuckoos were detected at 37 of 41 sites (90%) over the course of the study, with seven sites having detections only in 2019 and 16 sites having detections only in 2020 (Figure 1). Yellow‐billed Cuckoos were detected at 39 sites during the study (95%), with 14 sites having detections only in 2020 (Figure 1). For each species, we summarized differences in habitat and landscape characteristics at points and sites with at least one detection of a given species in 1 year, both years, and no years in Tables S2 and S3. In 2019, both species were detected at 16 and not detected at 11 sites (Odds Ratio = 3.91, 95% CI: 1.03–14.87), and in 2020 both were detected at 28 and not detected at 0 sites. We summarized differences in habitat and landscape characteristics at points and sites with at least one detection of both cuckoo species, one species, and no species in Table S4. We present the final model set from our dynamic, multi‐scale occupancy analysis for each species. All sub‐model sets are shown in Tables S5–S12.

### 3.1 Black‐billed Cuckoo habitat use and annual turnover

**FIGURE 2:**
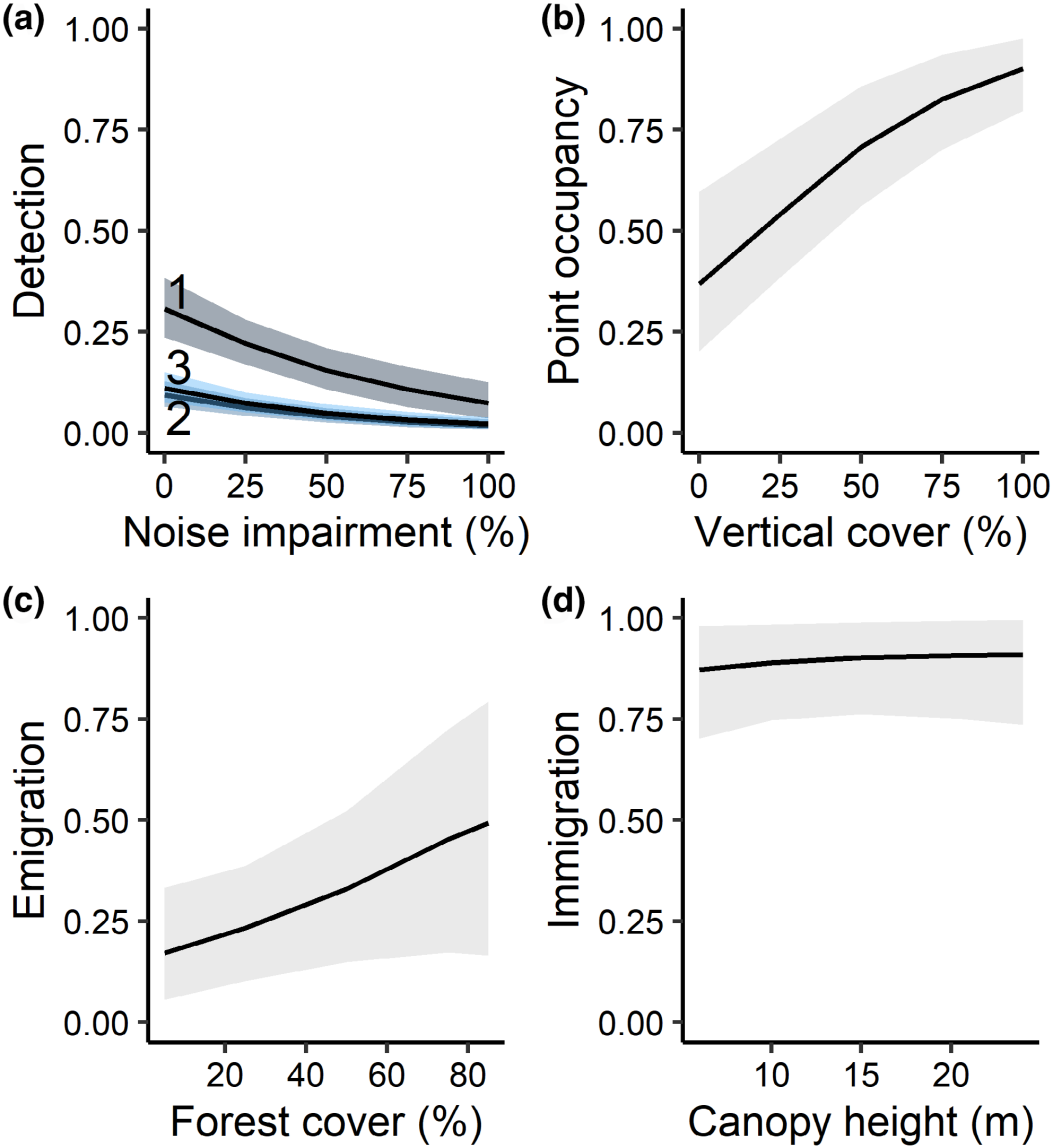
Predicted effects of covariates from the top Black‐billed Cuckoo occupancy model showing the effects of temporal variation (survey round) and noise on detection probability (a; number indicates round), vertical vegetation cover (≤2 m) on probability of point‐level use (b), forest cover in the surrounding landscape on extinction probability (c) and average canopy height at a site (>6 m) on colonization (d). The top initial site‐level occupancy structure was the constant model. Shown with 85% credible intervals.

The top detection structure for Black‐billed Cuckoos included additive effects of survey round and noise level (Table 1). Black‐billed Cuckoo detection probability was greatest at the beginning of the season and negatively impacted by noise (Table 2, Figure 2a). Under average conditions (hearing impaired during 0%–20% of survey time), the detection probability during the first survey round was 0.24 (85% Credible Interval calculated using MCMC [CRI]: 0.19, 0.30). However, the model including survey round and temperature was also supported in the detection sub‐model set (*w*_*i*_ = 0.37; Table S5). Initial site‐level occupancy was best predicted as constant (*ψ*_avg_ = 0.59, 85% CRI: 0.46, 0.72), though models including the proportion of developed area, crop cover, canopy cover, or open habitat were competitive (Table S6). The probability of point‐level use increased by 35% (85% CRI: 0.22, 0.49) for every 10% increase in vertical vegetation cover (Table 2, Figure 2b). Site occupancy increased from 0.59 in 2019 to 0.79 (85% CRI: 0.68, 0.89) in 2020.

**TABLE 1:** Candidate models describing between‐season Black‐billed Cuckoo and Yellow‐billed Cuckoo initial site‐level occupancy (*ψ*), point‐level use (*θ*), extinction (*ε*), colonization (*γ*), and detection probability (*p*).

Model	*k*	ΔAIC_c_	Dev	*w*_*i*_
Black‐billed Cuckoo				
*ψ*(.)*θ*(vertical cover)*ε*(forest)*γ*(canopy height)*p*(round + noise)	11	0.00^a^	836.54	0.26
*ψ*(.)*θ*(vertical cover)*ε*(forest)*γ*(.)*p*(round + noise)	10	0.18	839.39	0.24
*ψ*(.)*θ*(vertical cover)*ε*(forest)*γ*(open)p(round + noise)	11	0.34	836.88	0.22
*ψ*(.)*θ*(vertical cover)*ε*(forest)*γ*(vertical cover)*p*(round + noise)	11	0.89	837.42	0.17
*ψ*(.)*θ*(vertical cover)*ε*(forest)*γ*(forest)*p*(round + noise)	11	1.96	838.49	0.10
*ψ*(.)*θ*(vertical cover)*ε*(.)*γ*(.)*p*(round + noise)	9	6.91	848.72	0.01
***ψ(.)θ(vertical cover ε(0)γ(0)p(round + noise)***	***7***	***29.81***	***876.60***	***0.00***
Yellow‐billed Cuckoo				
*ψ*(development)*θ*(year + subcanopy height)*ε*(0)*γ*(canopy cover)*p*(year + temp)	10	0.00^b^	1701.71	0.90
*ψ*(development)*θ*(year + subcanopy height)*ε*(0)*γ*(.)*p*(year + temp)	9	4.41	1708.72	0.10
***ψ(development)θ(year + subcanopy height)ε(0)γ(0)p(year + temp)***	***8***	***26.54***	***1733.38***	***0.00***

*Note*: Models were ranked using Akaike's Information Criterion adjusted for small sample sizes (AIC_c_). We present ΔAIC_c_, the difference between the AIC_c_ score for a given model and the top model, *k*, the number of model parameters, model deviance (Dev), and model weight (*w*_*i*_). Black‐billed Cuckoo detection was modeled using additive effects of temporal variation (round) and noise while the Yellow‐billed Cuckoo model included additive effects of year and temperature (temp). Both non‐dynamic models received no support (bold/italic). Surveys were conducted in Illinois in 2019–2020.

^a^ AIC_c_ = 862.31.

^b^ AIC_c_ = 1724.81.

**TABLE 2:** Parameter estimates for the AIC_c_ best‐ranked Black‐billed Cuckoo (85% credible intervals) and Yellow‐billed Cuckoo (85% confidence intervals) occupancy models.

	Variable	Mean *β*	LCRI	UCRI
Black‐billed Cuckoo				
*ψ*	Intercept	0.39	−0.14	0.93
*θ*	Intercept	−0.54	−1.36	0.34
	Vertical cover	0.03	0.02	0.04
*ε*	Intercept	−1.81	−2.97	−0.73
	Forest	0.02	0.00	0.04
*γ*	Intercept	2.06	0.41	3.80
	Canopy height	0.04	−0.06	0.14
*p*	Round1	−0.82	−1.16	−0.46
	Round2	−2.29	−2.67	−1.90
	Round3	−2.10	−2.48	−1.73
	Noise	−0.45	−0.64	−0.27

	Variable	Mean *β*	LCI	UCI
Yellow‐billed Cuckoo				
*ψ*	Intercept	3.74	1.06	6.42
	Development	−0.10	−0.17	−0.03
*θ*	2019	0.77	0.12	1.42
	2020	2.74	1.78	3.69
	Subcanopy height	−0.32	−0.49	−0.15
*ε*	0	0.00	0.00	0.00
*γ*	Intercept	−11.91	−25.32	1.51
	Canopy cover	0.21	0.00	0.42
*p*	2019	−3.05	−3.60	−2.51
	2020	−2.74	−3.25	−2.22
	Temp	0.10	0.08	0.13

*Note*: Parameters include initial site‐level occupancy (*ψ*), point‐level use (*θ*), extinction (*ε*), colonization (*γ*), and detection probability (*p*). Black‐billed Cuckoo detection was modeled using additive effects of temporal variation (round) and noise while the Yellow‐billed Cuckoo detection model included additive effects of year and temperature (temp). Coefficient values (*β*) are on the original logit scale of each covariate.

We found strong support for Black‐billed Cuckoo site‐level turnover between years, as the top dynamic model was much more likely than the non‐dynamic model with ΔAIC_c_ = 29.81 (Table 1). In the top model, extinction probability increased by 22% (85% CRI: 0, 0.49) for every 10% increase in forest cover in the landscape (Table 2, Figure 2c); however, the credible interval contained 0. Colonization probability increased slightly with tree canopy height, with a 1 m increase making a site 4% (85% CRI: −0.06, 0.15) more likely to become occupied the following year, although the credible interval crossed 0 (Table 2, Figure 2d). The top five models in the final set, only varying in covariates modeling colonization, were within 2 ΔAIC_c_ units of the top model, indicating difficulty differentiating among them. The second model, which predicted colonization as constant (*γ*_avg_ = 0.88, 85% CRI: 0.74, 0.98) had a model weight of 0.24 (Table 1).

### 3.2 Yellow‐billed cuckoo habitat use and annual turnover

**FIGURE 3:**
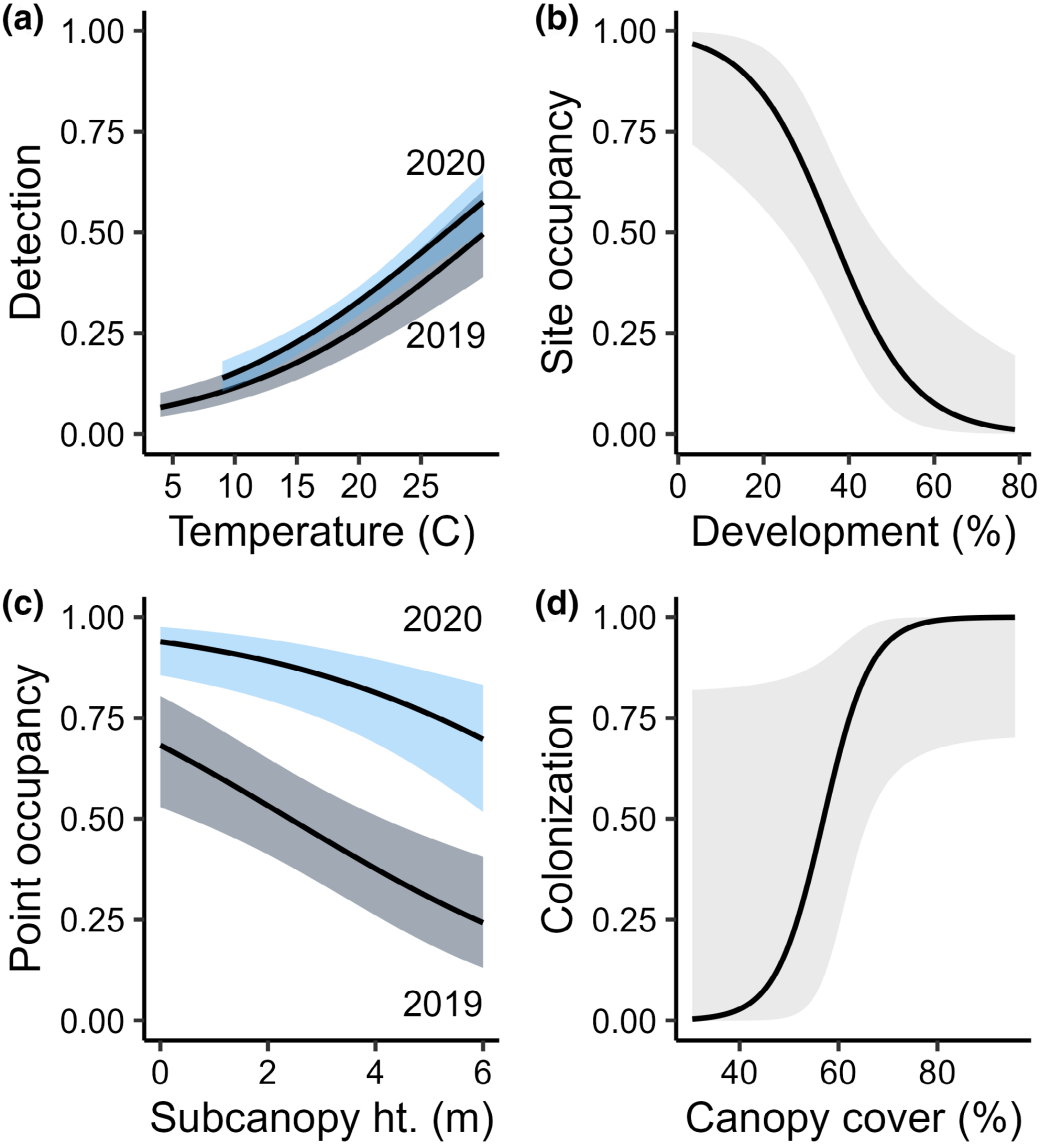
Predicted effects of covariates from the top Yellow‐billed Cuckoo occupancy model showing the effects of year and temperature on detection probability (a), the proportion of development in the surrounding landscape on initial site‐level occupancy probability (b), average subcanopy height at a site (woody vegetation ≤6 m) and year on the probability of point‐level use (c) and average canopy cover at a site on colonization (d). The extinction coefficient was held at 0 in the top model. Shown with 85% confidence intervals.

The best model structure for Yellow‐billed Cuckoo detection probability included the additive effects of year and temperature (Table 1). Detection was higher in 2020 than in 2019 and increased with temperature (Table 2, Figure 3a). Under average conditions (19.2°C, SE = 1.74), detection probability was 0.25 (85% CI: 0.19–0.31) in 2019 and 0.31 (85% CI: 0.28–0.35) in 2020. Initial site‐level occupancy probability decreased by 10% (85% CI: −0.16, −0.03) for every 1% increase in developed area in the surrounding landscape (Table 2, Figure 3b). The probability of point‐level use was greater in 2020 than in 2019 and decreased by 27% (85% CI: −0.39, −0.14) for every 1 m increase in subcanopy height (Table 2, Figure 3c). Estimated site‐level occupancy was 0.78 (85% CI: 0.58, 1) in 2019 and 0.99 (85% CI: 0.97, 1) in 2020.

We found substantial support for Yellow‐billed Cuckoo interannual site‐level turnover. The top dynamic model in the final model set was well supported (*w*_*i*_ = 0.90) while the non‐dynamic model ranked poorly (ΔAIC_c_ = 26.54; Table 1). Yellow‐billed Cuckoos were detected at all but 2 sites in 2020, and there were no sites with detections only in 2019 (Figure 1). Because no sites went extinct between 2019 and 2020, based on passive detections alone, we fixed extinction probability at 0 throughout the model selection process for this species. Colonization probability increased by 23% (85% CI: 0, 0.52) for every 1% increase in canopy cover (Table 2, Figure 3d), although canopy cover varied little at the site level (SE = 3.3%; Table S1) and the confidence interval contained 0.

## 4 DISCUSSION

We found strong support for Black‐billed and Yellow‐billed Cuckoo site‐level turnover between years, confirming prior observations (e.g., Halterman, 2009; Hughes, 2020b). As we predicted, Black‐billed Cuckoos were associated with a denser shrub layer within sites. While no variables were strongly associated with larger‐scale occupancy, Black‐billed Cuckoo persistence at sites in less forested landscapes suggests that larger areas of open habitat are important for this species. Yellow‐billed Cuckoos used patches with an open understory. This, their negative association with development, and colonization of sites with greater canopy cover point to a preference for patches and landscapes with dense tree cover. We also found that detection probability is the greatest during quiet conditions and early in the season for Black‐billed Cuckoos and during warmer conditions for Yellow‐billed Cuckoos.

Black‐billed Cuckoos' association with dense shrub patches is consistent with an Illinois study that concluded they are shrubland obligates (Robinson et al., 1999) and observations that Black‐billed Cuckoos tend to forage in shorter vegetation (table 1D in Johnson, 2021). Native shrubs support a diversity of potential insect prey (e.g., *Rhus* sp., a common plant at many of our sites; Fickenscher et al., 2014) and provide nest concealment. Thus, a dense shrub layer could increase foraging opportunities and decrease nest predation for Black‐billed Cuckoos. Yellow‐billed Cuckoos were more likely to use areas within sites that had a shorter, or even absent, subcanopy layer. Given how we defined subcanopy (woody vegetation ≤6 m), we interpreted this as a preference for a more open understory where woody vegetation was either short or had aged out of our subcanopy classification, given past research indicating preference for foraging in taller vegetation (>10 m; e.g., Laymon & Halterman, 1985) and selecting nest sites in dense stands of small trees with greater canopy cover (McNeil et al., 2013; Reiley & Benson, 2019). Wilson (1999) found Yellow‐billed Cuckoos had increased nest success in denser stands of trees, and greater canopy cover in these stands could also protect nests from temperature extremes (Burton, 2006). However, Yellow‐billed Cuckoos will nest in shorter, denser vegetation (Hughes, 2020a), making it important to provide areas of shrubby habitat for this species, as well as Black‐billed Cuckoos.

Yellow‐billed Cuckoos were less likely to occupy sites with more development in the landscape. Although no variables were particularly strong predictors of Black‐billed Cuckoo initial site‐level occupancy, there was some suggestion this species followed the same trend (development in the landscape was the second‐best predictor of site occupancy; Table S6). As open development by itself was highly correlated with low‐, medium‐, and high‐intensity development (*r* = .82), both species' negative relationship with our combined development variable is consistent with previous studies showing cuckoos have a negative relationship with developed green areas like mowed parks and golf courses (LeClerc & Cristol, 2005; Thogmartin & Knutson, 2007). Many atlases report Black‐billed and Yellow‐billed Cuckoos using urban green spaces (citations in Hughes, 2020a, 2020b) but it is likely birds are only more noticeable in these areas, not necessarily that they provide high‐quality habitat (LeClerc & Cristol, 2005).

We found that different variables predicted interannual turnover for each cuckoo species. Black‐billed Cuckoos persisted at sites with less forest in the landscape, although forest was a weak predictor. This is consistent with previous work that found a positive relationship with shrublands (Robinson et al., 1999) and that Black‐billed Cuckoos persisted at sites with more open habitat and colonized sites with less canopy cover (Johnson & Benson, 2022). This suggests that Black‐billed Cuckoos need large areas of shrubland habitat, perhaps to meet the energetic demands of large, fast‐developing chicks (Lack, 1968; Preble, 1957 cited by Wilson, 1999). However, Black‐billed Cuckoos' negative relationship with forest cover is contrary to findings from Thogmartin and Knutson (2007) in Wisconsin. This difference could be due to large differences in forest cover in Wisconsin and Illinois, or differences in scales used for analyses. Yellow‐billed Cuckoo colonization was more likely at sites with greater canopy cover, though again this was a weak predictor. Given their association with taller vegetation (Laymon & Halterman, 1985; McNeil et al., 2013; Wilson, 1999), forest cover in the landscape (Reiley & Benson, 2019), and negative association with shrub cover (Johnson & Benson, 2022), we believe this positive relationship with canopy cover is more strongly related to canopy cover from vegetation taller than the shrub layer. Similar to Black‐billed Cuckoos, Yellow‐billed Cuckoos may also need large areas of habitat, specifically forest, to have sufficient nesting and foraging opportunities to support offspring. Strong support for the top dynamic models demonstrated that accounting for interannual site‐level turnover is important for both cuckoo species. This is in contrast to most shrubland and forest species having relatively high‐site fidelity (Lehnen & Rodewald, 2009; Schlossberg, 2009). In addition McNeil et al. (2013) found Western Yellow‐billed Cuckoo site fidelity was fairly high in restored habitat, but habitat patches in the southwest are isolated, potentially making prospecting more costly (Teitelbaum et al., 2020).

The detection probability for each cuckoo species varied with different environmental factors. The decline in Black‐billed Cuckoo detection probability over the season could be attributed to a greater abundance of spring migrants during the first survey round (Royle & Nichols, 2003), as Johnson and Benson (2022) found detection was greatest mid‐season when accounting for within‐season dynamics. As such, performing surveys earlier in the season would be the best option to determine important breeding and stopover sites for this species. Yellow‐billed Cuckoo detection probability on the other hand increased with temperature. As we observed Yellow‐billed Cuckoos calling into the afternoon, waiting to start surveys, or limiting surveys to warmer days, would be best to determine the presence of this species. Yellow‐billed Cuckoo detection probability was also higher in 2020. As more sites *and* more points were occupied, greater detection probability could be due to an increase in abundance, and additionally tied to increased vocalization rates at greater densities (McShea & Rappole, 1997). Indeed, Reiley and Benson (2019) recorded highly variable Yellow‐billed Cuckoo abundances over 4 field seasons.

Although broadcast surveys increase detection, improving the estimation of habitat associations, we acknowledge that using broadcasts could bias estimates by attracting cuckoos away from used habitats different from that at a survey point. Nonetheless, we found that neither species moved significantly closer to observers between passive and broadcast surveys, consistent with the unaggressive behavior noted by Halterman (2009). In addition, given our systematic selection of points (i.e., selection from a random grid), vegetation plots should have been representative of the area surrounding a point. Broadcasts may also lead to concerns about non‐independence, as birds could follow observers to subsequent points. However, we did not detect either species at adjacent points more often (Black‐billed Cuckoos 17%, Yellow‐billed Cuckoos 24%) than expected by chance (32%), and neither species responded aggressively to broadcast, leading us to believe any non‐independence that was not accounted for was minimal. Furthermore, our modeling approach accounts for the non‐independence of points within sites (Kéry & Royle, 2016, 2021; Nichols et al., 2008).

While we found support for Black‐billed and Yellow‐billed Cuckoo interannual turnover, variables in the top colonization and extinction models were not strong predictors. As such, we believe other factors, like food abundance, could impact turnover rates. Others have postulated that these two species behave nomadically following spring migration, assessing resource availability before deciding where to breed (Hamilton & Hamilton, 1965; Nolan Jr. & Thompson, 1974), and more recent studies seem to confirm this. For example, Barber (2008) and Koenig and Liebhold (2005) both used Breeding Bird Survey data to show that the abundance of both cuckoo species increased significantly in areas with gypsy moth outbreaks and periodical cicada emergences, respectively, compared to years before and after. We collected data during non‐outbreak years, and still found evidence of high‐interannual turnover, potentially driven by smaller scale variation in insect availability. In addition, Johnson and Benson (2022) observed departures from sites well into the breeding season, including radio‐tagged Black‐billed Cuckoos with nests. However, we were not able to measure insect abundance and more work is needed to understand the ultimate and proximate cues determining when and where cuckoos decide to breed. In addition, the estimation of colonization and extinction parameters could be improved with additional years of data. Now that we have established the presence of interannual dynamics for both species, considering longer‐term datasets and examining insect availability could be the next steps toward understanding the movement ecology of Black‐billed and Yellow‐billed Cuckoos.

Given that Black‐billed and Yellow‐billed Cuckoos in this region are associated with shrubland and open forest habitat, respectively, loss of these habitat types has likely played a role in population declines. In addition to habitat destruction, the lack of historical disturbance regimes, primarily fire suppression, has resulted in open lands turning into closed forests (Nowacki & Abrams, 2008 and citations therein). The total forest area in Illinois has increased slightly since the early 1900s (following a 78% loss post‐European settlement), but oak‐hickory forests are steadily being replaced by more shade‐tolerant communities (Bretthauser & Edgington, 2003). In addition, invasive shrub cover has also continued to increase in Midwestern forests (Moser et al., 2016). Since 1950, over 60% of shrubland habitat has been lost in the Central Hardwoods region (King & Schlossberg, 2014) and prior to that, orchard and hedgerow acreage in Illinois, specifically, declined by 90% between 1909 and 1958 (Graber & Graber, 1963). There was a peak in shrubland availability in the early 20th century when large areas of farmland were abandoned. However, indigenous peoples maintained large tracts of open habitat prior to European colonization and agricultural expansion (Askins, 1994), meaning these declines are not just a return to baseline. Within‐site management that creates or maintains patches of short shrubby vegetation and open woodlands in landscapes with less development, would benefit both cuckoo species. Black‐billed Cuckoos in particular would benefit from management in landscapes with less forest cover. However, given strong evidence of annual dynamics, coordinating management at a broader spatial scale to provide a shifting mosaic of suitable habitat will be equally important (Runge et al., 2014; Woinarski et al., 1992).

We used dynamic multi‐scale occupancy models to establish that Black‐billed and Yellow‐billed Cuckoos occupy different locations from year to year. The presence of occupancy dynamics has significant implications for habitat management and conservation. Assuming distributions are static and that preferred habitat is always used (Battin, 2004) could lead to misinformed decisions if a species occupies different areas depending on the season, year, or even longer cycles (Green et al., 2018; Pendleton et al., 2022; Webb et al., 2017). This makes dynamic models a useful tool for understanding habitat requirements and movement ecology of highly mobile species, like birds (Tingley et al., 2018), as well as general availability processes for more sedentary species like reptiles (e.g., tortoises that cannot be detected in burrows; Harju & Cambrin, 2019), and even plants (e.g., absence of above‐ground parts in a particular season; Gray et al., 2013). The model's requirement for simple detection/non‐detection data also means it can be applied to rare and difficult to capture species. Extending the dynamic model to consider multiple scales (Green et al., 2018) allowed us to account for the possibility of scale‐specific dependencies (Chiavacci et al., 2018; Hagen et al., 2016). Examining habitat requirements at both small and large scales can provide relevant guidance to both site managers, wishing to create suitable habitat for a given species, and organizations acquiring land, which must prioritize selecting parcels in the best landscape context in the face of limited funds (Duren et al., 2011).

## AUTHOR CONTRIBUTIONS

**Claire A. Johnson:** Conceptualization (equal); formal analysis (equal); investigation (lead); methodology (lead); supervision (equal); writing – original draft (equal). **Thomas J. Benson:** Conceptualization (equal); formal analysis (equal); investigation (supporting); methodology (supporting); supervision (equal); writing – original draft (equal).

## CONFLICT OF INTEREST STATEMENT

The authors have no competing interests to disclose.

## Supporting information


Tables S1–S12.

## Data Availability

Analyses reported in this article can be reproduced using the data provided by Johnson and Benson (2023).
