# Supplementary material for: Dynamic, multi‐scale analyses indicate site‐ and landscape‐level forest cover drive Yellow‐billed and Black‐billed Cuckoo interannual turnover
Source: Ecol Evol. 2024 Feb 7;14(2):e10938. doi: 10.1002/ece3.10938 (PMC10850813; doi:10.1002/ece3.10938)
Supplement: Supplementary file 1 — Tables S1–S12. [file ECE3-14-e10938-s001.docx]

**Table S1.** Covariates used in Black-billed and Yellow-billed Cuckoo occupancy models. Point-level environmental and vegetation variables were used to model detection and point-level use. Vegetation variables, averaged by site, and the proportion of landcover classes within 700 m of each transect were used to model initial site-level occupancy, colonization, and extinction.

|  | Variable | Mean | SE | Min. | Max. |  | Mean | SE | Min. | Max. |
| --- | --- | --- | --- | --- | --- | --- | --- | --- | --- | --- |
| Point |  |  |  |  |  | Site |  |  |  |  |
|  | Cloud Cover | 2.27 | 0.51 | 1 | 4 |  |  |  |  |  |
|  | Wind | 1.13 | 0.46 | 0 | 6 |  |  |  |  |  |
|  | Temperature (°C) | 19.20 | 1.74 | 4 | 30 |  |  |  |  |  |
|  | Noise | 0.81 | 0.35 | 0 | 4 |  |  |  |  |  |
|  | Small Trees (8-23 cm DBH) | 7.76 | 2.04 | 0 | 28 |  | 7.72 | 0.76 | 3.00 | 14.44 |
|  | Medium Trees (23-38 cm DBH) | 2.16 | 0.70 | 0 | 13 |  | 2.11 | 0.27 | 0.43 | 5.33 |
|  | Canopy Cover (%) | 73.29 | 9.22 | 0 | 99.48 |  | 73.26 | 3.31 | 30.42 | 95.68 |
|  | Canopy Height (m) | 14.80 | 2.30 | 0 | 34.44 |  | 14.57 | 1.03 | 6.01 | 24.03 |
|  | Subcanopy Height (m) | 2.16 | 0.51 | 0 | 6 |  | 2.19 | 0.23 | 0.17 | 4.41 |
|  | Shrub Cover (%) | 17.34 | 5.28 | 0 | 83.50 |  | 17.48 | 2.17 | 4.00 | 49.44 |
|  | Brush Cover (%) | 3.05 | 1.46 | 0 | 33.75 |  | 3.12 | 0.59 | 0.10 | 11.95 |
|  | Vertical Vegetation Cover (%) | 49.25 | 7.74 | 1.56 | 99.56 |  | 49.79 | 3.32 | 16.02 | 71.89 |
|  | Developed Area (%) |  |  |  |  |  | 23.58 | 4.70 | 3.28 | 78.91 |
|  | Forest (%) |  |  |  |  |  | 38.57 | 4.26 | 3.59 | 84.87 |
|  | Cultivated Crops (%) |  |  |  |  |  | 10.93 | 3.32 | 0.00 | 54.23 |
|  | Open Habitat (%) |  |  |  |  |  | 15.41 | 2.91 | 0.10 | 46.73 |

**Table S2.** Mean (and standard deviation) of habitat variables at points with a Black-billed Cuckoo (BBCU) or Yellow-billed Cuckoo (YBCU) detection in neither 2019 or 2020 (none), just 1 year (one), or both years.

|  | BBCU | | | | YBCU | | |
| --- | --- | --- | --- | --- | --- | --- | --- |
|  | None (N=237) | | One (N=95) | Both (N=12) | None (N=134) | One (N=146) | Both (N=64) |
| Small Trees (8-23 cm DBH) | | 7.94 (6.27) | 7.78 (6.96) | 4.17 (5.29) | 7.32 (6.17) | 8.73 (6.83) | 6.47 (5.9) |
| Medium Trees (23-38 cm DBH) | | 2.38 (2.2) | 1.83 (2.3) | 1 (1.48) | 1.99 (1.98) | 2.32 (2.34) | 2.25 (2.44) |
| Canopy Cover (%) | | 76.28 (26.87) | 69.58 (31.7) | 51.61 (35.74) | 72.59 (29.34) | 76.38 (28.43) | 69.21 (29.21) |
| Canopy Height (m) | | 15.56 (6.92) | 13.34 (7.9) | 12.09 (6.61) | 13.93 (7.32) | 15.38 (7.08) | 15.45 (7.46) |
| Subcanopy Height (m) | | 2.08 (1.68) | 2.41 (1.46) | 2.01 (1.58) | 2.38 (1.67) | 2.21 (1.55) | 1.62 (1.59) |
| Vertical Vegetation Cover (%) | | 44.94 (24.37) | 59.2 (22.46) | 53.22 (29.16) | 49.64 (24.21) | 49.02 (25.35) | 48.54 (25.1) |
| Shrub Cover (%) | | 16.66 (16.26) | 18.97 (17.61) | 15.04 (11.37) | 19.34 (18.43) | 16.24 (14.26) | 15.13 (16.76) |
| Brush Cover (%) | | 3.08 (4.49) | 3.23 (5.44) | 1.71 (2.38) | 2.9 (4.96) | 3.52 (4.65) | 2.43 (4.25) |

**Table S3.** Mean (and standard deviation) of site-averaged habitat and landscape variables at sites with a Black-billed Cuckoo (BBCU) or Yellow-billed Cuckoo (YBCU) detection in neither 2019 or 2020 (none), only 1 year (one), or both years.

|  | BBCU | | | YBCU | | |
| --- | --- | --- | --- | --- | --- | --- |
|  | None (N=4) | One (N=23) | Both (N=14) | None (N=2) | One (N=14) | Both (N=25) |
| Small Trees (8-23 cm DBH) | 7.55 (2.95) | 8.04 (3.45) | 7.19 (2.78) | 4.68 (0.76) | 7.58 (3.58) | 8.02 (2.93) |
| Medium Trees (23-38 cm DBH) | 2.46 (0.85) | 2.28 (1.26) | 1.76 (0.91) | 1.48 (0.27) | 2.09 (1.37) | 2.19 (1.02) |
| Canopy Cover (%) | 83.61 (9.34) | 76.16 (9.3) | 65.8 (17.36) | 52.74 (4.56) | 72.79 (8.53) | 75.31 (15.22) |
| Canopy Height (m) | 17.17 (7.12) | 14.33 (4.28) | 14.25 (3.33) | 10.98 (0.47) | 13.3 (3.57) | 15.58 (4.52) |
| Subcanopy Height (m) | 1.79 (0.41) | 2.23 (0.9) | 2.27 (1.15) | 3.13 (0.2) | 2.13 (1.07) | 2.16 (0.91) |
| Vertical Vegetation Cover (%) | 40.32 (22.57) | 49.65 (12.37) | 52.5 (13.15) | 48.94 (17.35) | 47.8 (12.37) | 50.85 (14.76) |
| Shrub Cover (%) | 17.63 (11.95) | 17.54 (10.13) | 17.44 (6.2) | 34.67 (20.9) | 16.42 (8.11) | 16.75 (7.29) |
| Brush Cover (%) | 3.62 (2.05) | 3.33 (2.72) | 2.68 (2.27) | 1.42 (0.82) | 3.54 (2.28) | 3.04 (2.66) |
| Developed Area (%) | 31.61 (9.62) | 23.75 (17.12) | 21 (24.71) | 62.8 (22.78) | 31.97 (19.42) | 15.75 (12.91) |
| Forest (%) | 49.79 (12.67) | 42.17 (16.84) | 29.44 (16.74) | 22.13 (12.55) | 38.62 (12.35) | 39.85 (20.01) |
| Cultivated Crops (%) | 2.8 (3.51) | 10.97 (13.9) | 13.19 (14.9) | 1.33 (1.68) | 8.38 (12.12) | 13.13 (14.73) |
| Open Habitat (%) | 10.1 (9.58) | 13.36 (7.76) | 20.31 (16.68) | 5.65 (6.9) | 12.28 (5.29) | 17.95 (14.23) |

**Table S4.** Mean (and standard deviation) of point-level, site-averaged habitat, and landscape variables at points and sites with a detection of at least one Black-billed Cuckoo (BBCU) and at least one Yellow-billed Cuckoo (YBCU) in the same year (both), just one species (one), and where neither species was detected (none). All sites had at least one of the species present in both years.

|  | Site | | Point | | |
| --- | --- | --- | --- | --- | --- |
|  | One (N=7) | Both (N=34) | None (N=103) | One (N=181) | Both (N=60) |
| Small Trees (8-23 cm DBH) | 7.17 (2.93) | 7.82 (3.21) | 7.82 (6.13) | 7.77 (6.53) | 7.63 (6.84) |
| Medium Trees (23-38 cm DBH) | 2.22 (0.8) | 2.1 (1.19) | 2.2 (2.08) | 2.34 (2.35) | 1.65 (2.03) |
| Canopy Cover (%) | 74.28 (16.29) | 73.16 (13.35) | 76.23 (26.31) | 72.65 (30.22) | 71.79 (29.65) |
| Canopy Height (m) | 15.41 (5.88) | 14.41 (3.96) | 14.82 (7.1) | 15.08 (7.16) | 14.08 (7.89) |
| Subcanopy Height (m) | 2.16 (0.73) | 2.21 (1) | 2.25 (1.74) | 2.12 (1.56) | 2.17 (1.61) |
| Vertical Vegetation Cover (%) | 43.18 (17.93) | 51.06 (12.69) | 46.47 (23.41) | 47.8 (25.67) | 57.93 (22.79) |
| Shrub Cover (%) | 23.48 (14.45) | 16.29 (7.04) | 18.65 (18.37) | 16.99 (15.52) | 15.59 (16.08) |
| Brush Cover (%) | 2.96 (1.83) | 3.17 (2.62) | 2.96 (5.07) | 3.18 (4.31) | 2.95 (5.3) |
| Developed Area (%) | 37.09 (22.76) | 20.8 (17.72) |  |  |  |
| Forest (%) | 42.16 (17.15) | 37.83 (17.8) |  |  |  |
| Cultivated Crops (%) | 5.55 (8.98) | 12.04 (14.34) |  |  |  |
| Open Habitat (%) | 9.64 (8.09) | 16.6 (12.41) |  |  |  |

In all detection sub-models (p) we considered effects of temporal variation (round), year, temperature (temp), noise, wind speed, and cloud cover. For initial site-level occupancy (ψ) we considered the number of small and medium trees (med tree), total canopy cover, average maximum canopy height and subcanopy height, vertical vegetation cover (<2m), shrub cover, and brush cover, averaged across the entire site, and additionally included the proportion of the 700 m transect buffer covered by developed land (combined open, low, medium and high development), forest (combined deciduous, evergreen, mixed forest, and woody wetlands), cultivated crops (agriculture), and open habitat (habitat herbaceous and hay/pasture) as defined in the National Land Cover Dataset. Point-level use (θ) sub-models included effects of year and the same 8 habitat variables, measured at each point. Extinction (ε) and colonization (γ) were modelled as a function of site-level habitat and landscape variables.

**Table S5.** Candidate models describing Black-billed Cuckoo detection probability.

| Model | *k* | AIC_c_ | ΔAIC_c_ | Dev | *w_i_* |
| --- | --- | --- | --- | --- | --- |
| ψ(.)θ(.)ε(.)γ(.)p(round+noise) | 8 | 880.03 | 0.00 | 862.06 | 0.62 |
| ψ(.)θ(.)ε(.)γ(.)p(round+temp) | 8 | 881.04 | 1.01 | 863.07 | 0.37 |
| ψ(.)θ(.)ε(.)γ(.)p(round) | 7 | 890.88 | 10.85 | 875.37 | 0.00 |
| ψ(.)θ(.)ε(.)γ(.)p(round+wind) | 8 | 892.63 | 12.60 | 874.66 | 0.00 |
| ψ(.)θ(.)ε(.)γ(.)p(round+cloud) | 8 | 892.86 | 12.82 | 874.88 | 0.00 |
| ψ(.)θ(.)ε(.)γ(.)p(.) | 5 | 932.23 | 52.20 | 921.44 | 0.00 |
| ψ(.)θ(.)ε(.)γ(.)p(year) | 6 | 935.01 | 54.98 | 921.89 | 0.00 |

**Table S6.** Candidate models describing Black-billed Cuckoo initial site-level occupancy probability.

| Model | *k* | AIC_c_ | ΔAIC_c_ | Dev | *w_i_* |
| --- | --- | --- | --- | --- | --- |
| ψ(.)θ(.)ε(.)γ(.)p(round+noise) | 8 | 880.03 | 0.00 | 862.06 | 0.16 |
| ψ(development)θ(.)ε(.)γ(.)p(round+noise) | 9 | 880.20 | 0.16 | 859.70 | 0.15 |
| ψ(agriculture)θ(.)ε(.)γ(.)p(round+noise) | 9 | 880.95 | 0.92 | 860.45 | 0.10 |
| ψ(canopy cover)θ(.)ε(.)γ(.)p(round+noise) | 9 | 881.06 | 1.03 | 860.56 | 0.10 |
| ψ(open)θ(.)ε(.)γ(.)p(round+noise) | 9 | 881.18 | 1.15 | 860.68 | 0.09 |
| ψ(brush)θ(.)ε(.)γ(.)p(round+noise) | 9 | 882.05 | 2.02 | 861.55 | 0.06 |
| ψ(forest)θ(.)ε(.)γ(.)p(round+noise) | 9 | 882.16 | 2.13 | 861.66 | 0.06 |
| ψ(small tree)θ(.)ε(.)γ(.)p(round+noise) | 9 | 882.17 | 2.14 | 861.67 | 0.05 |
| ψ(med tree)θ(.)ε(.)γ(.)p(round+noise) | 9 | 882.37 | 2.34 | 861.87 | 0.05 |
| ψ(vertical cover)θ(.)ε(.)γ(.)p(round+noise) | 9 | 882.41 | 2.38 | 861.91 | 0.05 |
| ψ(shrub)θ(.)ε(.)γ(.)p(round+noise) | 9 | 882.44 | 2.41 | 861.94 | 0.05 |
| ψ(canopy height)θ(.)ε(.)γ(.)p(round+noise) | 9 | 882.55 | 2.52 | 862.05 | 0.05 |
| ψ(subcanopy height)θ(.)ε(.)γ(.)p(round+noise) | 9 | 882.56 | 2.53 | 862.06 | 0.05 |

**Table S7.** Candidate models describing Black-billed Cuckoo point-level use probability.

| Model | *k* | AIC_c_ | ΔAIC_c_ | Dev | *w_i_* |
| --- | --- | --- | --- | --- | --- |
| ψ(.)θ(vertical cover)ε(.)γ(.)p(round+noise) | 9 | 869.22 | 0.00 | 848.72 | 0.91 |
| ψ(.)θ(subcanopy height)ε(.)γ(.)p(round+noise) | 9 | 875.25 | 6.03 | 854.75 | 0.04 |
| ψ(.)θ(shrub)ε(.)γ(.)p(round+noise) | 9 | 877.15 | 7.93 | 856.65 | 0.02 |
| ψ(.)θ(canopy coverer)ε(.)γ(.)p(round+noise) | 9 | 878.69 | 9.47 | 858.19 | 0.01 |
| ψ(.)θ(canopy height)ε(.)γ(.)p(round+noise) | 9 | 879.05 | 9.83 | 858.55 | 0.01 |
| ψ(.)θ(.)ε(.)γ(.)p(round+noise) | 8 | 880.03 | 10.81 | 862.06 | 0.00 |
| ψ(.)θ(med tree)ε(.)γ(.)p(round+noise) | 9 | 880.66 | 11.44 | 860.16 | 0.00 |
| ψ(.)θ(year)ε(.)γ(.)p(round+noise) | 9 | 881.66 | 12.44 | 861.16 | 0.00 |
| ψ(.)θ(small tree)ε(.)γ(.)p(round+noise) | 9 | 882.42 | 13.19 | 861.92 | 0.00 |
| ψ(.)θ(brush)ε(.)γ(.)p(round+noise) | 9 | 882.56 | 13.34 | 862.06 | 0.00 |

**Table S8.** Candidate models describing Black-billed Cuckoo extinction probability.

| Model | *k* | AIC_c_ | ΔAIC_c_ | Dev | *w_i_* |
| --- | --- | --- | --- | --- | --- |
| ψ(.)θ(vertical cover)ε(forest)γ(.)p(round+noise) | 10 | 862.49 | 0.00 | 839.39 | 0.74 |
| ψ(.)θ(vertical cover)ε(canopy cover)γ(.)p(round+noise) | 10 | 866.57 | 4.08 | 843.47 | 0.10 |
| ψ(.)θ(vertical cover)ε(med tree)γ(.)p(round+noise) | 10 | 868.10 | 5.61 | 845.00 | 0.04 |
| ψ(.)θ(vertical cover)ε(.)γ(.)p(round+noise) | 9 | 869.22 | 6.73 | 848.72 | 0.03 |
| ψ(.)θ(vertical cover)ε(canopy height)γ(.)p(round+noise) | 10 | 869.78 | 7.29 | 846.68 | 0.02 |
| ψ(.)θ(vertical cover)ε(open)γ(.)p(round+noise) | 10 | 870.22 | 7.74 | 847.12 | 0.02 |
| ψ(.)θ(vertical cover)ε(vertical cover)γ(.)p(round+noise) | 10 | 870.59 | 8.10 | 847.49 | 0.01 |
| ψ(.)θ(vertical cover)ε(small tree)γ(.)p(round+noise) | 10 | 871.25 | 8.76 | 848.15 | 0.01 |
| ψ(.)θ(vertical cover)ε(agriculture)γ(.)p(round+noise) | 10 | 871.33 | 8.85 | 848.24 | 0.01 |
| ψ(.)θ(vertical cover)ε(development)γ(.)p(round+noise) | 10 | 871.42 | 8.93 | 848.32 | 0.01 |
| ψ(.)θ(vertical cover)ε(brush)γ(.)p(round+noise) | 10 | 871.54 | 9.05 | 848.44 | 0.01 |
| ψ(.)θ(vertical cover)ε(shrub)γ(.)p(round+noise) | 10 | 871.70 | 9.21 | 848.60 | 0.01 |
| ψ(.)θ(vertical cover)ε(subcanopy height)γ(.)p(round+noise) | 10 | 871.73 | 9.24 | 848.63 | 0.01 |

**Table S8.** Candidate models describing Black-billed Cuckoo colonization probability.

| Model | *k* | AIC_c_ | ΔAIC_c_ | Dev | *w_i_* |
| --- | --- | --- | --- | --- | --- |
| ψ(.)θ(vertical cover)ε(.)γ(canopy height)p(round+noise) | 10 | 868.48 | 0.00 | 845.38 | 0.19 |
| ψ(.)θ(vertical cover)ε(.)γ(open)p(round+noise) | 10 | 868.63 | 0.15 | 845.53 | 0.18 |
| ψ(.)θ(vertical cover)ε(.)γ(.)p(round+noise) | 9 | 869.22 | 0.74 | 848.72 | 0.13 |
| ψ(.)θ(vertical cover)ε(.)γ(vertical cover)p(round+noise) | 10 | 869.55 | 1.07 | 846.45 | 0.11 |
| ψ(.)θ(vertical cover)ε(.)γ(forest)p(round+noise) | 10 | 870.20 | 1.72 | 847.10 | 0.08 |
| ψ(.)θ(vertical cover)ε(.)γ(canopy cover)p(round+noise) | 10 | 871.13 | 2.65 | 848.03 | 0.05 |
| ψ(.)θ(vertical cover)ε(.)γ(agriculture)p(round+noise) | 10 | 871.30 | 2.82 | 848.20 | 0.05 |
| ψ(.)θ(vertical cover)ε(.)γ(med tree)p(round+noise) | 10 | 871.82 | 3.34 | 848.72 | 0.04 |
| ψ(.)θ(vertical cover)ε(.)γ(small tree)p(round+noise) | 10 | 871.82 | 3.34 | 848.72 | 0.04 |
| ψ(.)θ(vertical cover)ε(.)γ(subcanopy height)p(round+noise) | 10 | 871.82 | 3.34 | 848.72 | 0.04 |
| ψ(.)θ(vertical cover)ε(.)γ(shrub)p(round+noise) | 10 | 871.82 | 3.34 | 848.72 | 0.04 |
| ψ(.)θ(vertical cover)ε(.)γ(brush)p(round+noise) | 10 | 871.82 | 3.34 | 848.72 | 0.04 |
| ψ(.)θ(vertical cover)ε(.)γ(development)p(round+noise) | 10 | 871.82 | 3.34 | 848.72 | 0.04 |

**Table S9.** Candidate models describing Yellow-billed Cuckoo detection probability.

| Model | *k* | AIC_c_ | ΔAIC_c_ | Dev | *w_i_* |
| --- | --- | --- | --- | --- | --- |
| ψ(.)θ(.)ε(0)γ(.)p(year+temp) | 6 | 1749.63 | 0.00 | 1736.51 | 0.97 |
| ψ(.)θ(.)ε(0)γ(.)p(year+noise) | 6 | 1756.90 | 7.27 | 1743.78 | 0.03 |
| ψ(.)θ(.)ε(0)γ(.)p(year+cloud) | 6 | 1775.31 | 25.68 | 1762.19 | 0.00 |
| ψ(.)θ(.)ε(0)γ(.)p(year+wind) | 6 | 1782.09 | 32.47 | 1768.97 | 0.00 |
| ψ(.)θ(.)ε(0)γ(.)p(year) | 5 | 1782.28 | 32.66 | 1771.49 | 0.00 |
| ψ(.)θ(.)ε(0)γ(.)p(round) | 6 | 1791.97 | 42.34 | 1778.85 | 0.00 |
| ψ(.)θ(.)ε(0)γ(.)p(.) | 4 | 1821.47 | 71.84 | 1812.95 | 0.00 |

**Table S10.** Candidate models describing Yellow-billed Cuckoo initial site-level occupancy probability.

| Model | *k* | AIC_c_ | ΔAIC_c_ | Dev | *w_i_* |
| --- | --- | --- | --- | --- | --- |
| ψ(development)θ(.)ε(0)γ(.)p(year+temp) | 7 | 1738.62 | 0.00 | 1723.11 | 0.97 |
| ψ(canopy height)θ(.)ε(0)γ(.)p(year+temp) | 7 | 1748.56 | 9.94 | 1733.04 | 0.01 |
| ψ(open)θ(.)ε(0)γ(.)p(year+temp) | 7 | 1749.06 | 10.44 | 1733.54 | 0.01 |
| ψ(agriculture)θ(.)ε(0)γ(.)p(year+temp) | 7 | 1749.35 | 10.73 | 1733.83 | 0.00 |
| ψ(.)θ(.)ε(0)γ(.)p(year+temp) | 6 | 1749.63 | 11.01 | 1736.51 | 0.00 |
| ψ(canopy cover)θ(.)ε(0)γ(.)p(year+temp) | 7 | 1750.57 | 11.95 | 1735.06 | 0.00 |
| ψ(small tree)θ(.)ε(0)γ(.)p(year+temp) | 7 | 1751.11 | 12.49 | 1735.60 | 0.00 |
| ψ(shrub)θ(.)ε(0)γ(.)p(year+temp) | 7 | 1751.31 | 12.69 | 1735.80 | 0.00 |
| ψ(forest)θ(.)ε(0)γ(.)p(year+temp) | 7 | 1751.66 | 13.04 | 1736.15 | 0.00 |
| ψ(med tree)θ(.)ε(0)γ(.)p(year+temp) | 7 | 1751.66 | 13.05 | 1736.15 | 0.00 |
| ψ(subcanopy height)θ(.)ε(0)γ(.)p(year+temp) | 7 | 1751.70 | 13.08 | 1736.18 | 0.00 |
| ψ(vertical cover)θ(.)ε(0)γ(.)p(year+temp) | 7 | 1751.76 | 13.15 | 1736.25 | 0.00 |
| ψ(brush)θ(.)ε(0)γ(.)p(year+temp) | 7 | 1751.98 | 13.36 | 1736.46 | 0.00 |

**Table S11.** Candidate models describing Yellow-billed Cuckoo point-level use probability.

| Model | *k* | AIC_c_ | ΔAIC_c_ | Dev | *w_i_* |
| --- | --- | --- | --- | --- | --- |
| ψ(development)θ(year+subcanopy height)ε(0)γ(.)p(year+temp) | 9 | 1729.22 | 0.00 | 1708.72 | 0.84 |
| ψ(development)θ(year)ε(0)γ(.)p(year+temp) | 8 | 1735.34 | 6.12 | 1717.37 | 0.04 |
| ψ(development)θ(year+small tree)ε(0)γ(.)p(year+temp) | 9 | 1736.39 | 7.17 | 1715.89 | 0.02 |
| ψ(development)θ(year+canopy cover)ε(0)γ(.)p(year+temp) | 9 | 1736.59 | 7.38 | 1716.09 | 0.02 |
| ψ(development)θ(year+canopy height)ε(0)γ(.)p(year+temp) | 9 | 1737.05 | 7.84 | 1716.55 | 0.02 |
| ψ(development)θ(year+med tree)ε(0)γ(.)p(year+temp) | 9 | 1737.05 | 7.84 | 1716.55 | 0.02 |
| ψ(development)θ(year+vertical cover)ε(0)γ(.)p(year+temp) | 9 | 1737.28 | 8.06 | 1716.78 | 0.01 |
| ψ(development)θ(year+brush)ε(0)γ(.)p(year+temp) | 9 | 1737.54 | 8.32 | 1717.04 | 0.01 |
| ψ(development)θ(year+shrub)ε(0)γ(.)p(year+temp) | 9 | 1737.76 | 8.54 | 1717.26 | 0.01 |
| ψ(development)θ(.)ε(0)γ(.)p(year+temp) | 7 | 1738.62 | 9.40 | 1723.10 | 0.01 |

**Table S12.** Candidate models describing Yellow-billed Cuckoo colonization probability.

| Model | *k* | AIC_c_ | ΔAIC_c_ | Dev | *w_i_* |
| --- | --- | --- | --- | --- | --- |
| ψ(development)θ(year+subcanopy height)ε(0)γ(canopy cover)p(year+temp) | 10 | 1724.81 | 0.00 | 1701.71 | 0.46 |
| ψ(development)θ(year+subcanopy height)ε(0)γ(shrub)p(year+temp) | 10 | 1727.90 | 3.08 | 1704.80 | 0.10 |
| ψ(development)θ(year+subcanopy height)ε(0)γ(development)p(year+temp) | 10 | 1728.17 | 3.36 | 1705.07 | 0.09 |
| ψ(development)θ(year+subcanopy height)ε(0)γ(brush)p(year+temp) | 10 | 1728.95 | 4.14 | 1705.85 | 0.06 |
| ψ(development)θ(year+subcanopy height)ε(0)γ(small tree)p(year+temp) | 10 | 1729.17 | 4.36 | 1706.07 | 0.05 |
| ψ(development)θ(year+subcanopy height)ε(0)γ(.)p(year+temp) | 9 | 1729.22 | 4.41 | 1708.72 | 0.05 |
| ψ(development)θ(year+subcanopy height)ε(0)γ(forest)p(year+temp) | 10 | 1729.23 | 4.42 | 1706.13 | 0.05 |
| ψ(development)θ(year+subcanopy height)ε(0)γ(open)p(year+temp) | 10 | 1729.92 | 5.11 | 1706.82 | 0.04 |
| ψ(development)θ(year+subcanopy height)ε(0)γ(subcanopy height)p(year+temp) | 10 | 1730.20 | 5.39 | 1707.11 | 0.03 |
| ψ(development)θ(year+subcanopy height)ε(0)γ(canopy height)p(year+temp) | 10 | 1730.24 | 5.43 | 1707.14 | 0.03 |
| ψ(development)θ(year+subcanopy height)ε(0)γ(agriculture)p(year+temp) | 10 | 1731.13 | 6.32 | 1708.03 | 0.02 |
| ψ(development)θ(year+subcanopy height)ε(0)γ(med tree)p(year+temp) | 10 | 1731.14 | 6.33 | 1708.04 | 0.02 |
| ψ(development)θ(year+subcanopy height)ε(0)γ(vertical cover)p(year+temp) | 10 | 1731.80 | 6.98 | 1708.70 | 0.01 |
| ψ(development)θ(year+subcanopy height)ε(0)γ(0)p(year+temp) | 8 | 1751.35 | 26.54 | 1733.38 | 0.00 |
